# Tribbles homolog 3-mediated targeting the AKT/mTOR axis in mice with retinal degeneration

**DOI:** 10.1038/s41419-021-03944-w

**Published:** 2021-07-02

**Authors:** Irina V. Saltykova, Asif Elahi, Priyam M. Pitale, Oleg S. Gorbatyuk, Mohammad Athar, Marina S. Gorbatyuk

**Affiliations:** 1 Department of Optometry and Vision Science, School of Optometry, University of Alabama at Birmingham, Birmingham, AL USA; 2 Department of Dermatology, School of Medicine, University of Alabama at Birmingham, Birmingham, AL USA

**Keywords:** Translation, Diseases

## Abstract

Various retinal degenerative disorders manifest in alterations of the AKT/mTOR axis. Despite this, consensus on the therapeutic targeting of mTOR in degenerating retinas has not yet been achieved. Therefore, we investigated the role of AKT/mTOR signaling in *rd16* retinas, in which we restored the AKT/mTOR axis by genetic ablation of pseudokinase TRB3, known to inhibit phosphorylation of AKT and mTOR. First, we found that TRB3 ablation resulted in preservation of photoreceptor function in degenerating retinas. Then, we learned that the mTOR downstream cellular pathways involved in the homeostasis of photoreceptors were also reprogrammed in *rd16* TRB3^−/−^ retinas. Thus, the level of inactivated translational repressor p-4E-BP1 was significantly increased in these mice along with the restoration of translational rate. Moreover, in *rd16* mice manifesting decline in p-mTOR at P15, we found elevated expression of Beclin-1 and ATG5 autophagy genes. Thus, these mice showed impaired autophagy flux measured as an increase in LC3 conversion and p62 accumulation. In addition, the RFP-EGFP-LC3 transgene expression in *rd16* retinas resulted in statistically fewer numbers of red puncta in photoreceptors, suggesting impaired late autophagic vacuoles. In contrast, TRIB3 ablation in these mice resulted in improved autophagy flux. The restoration of translation rate and the boost in autophagosome formation occurred concomitantly with an increase in total Ub and rhodopsin protein levels and the elevation of E3 ligase Parkin1. We propose that TRB3 may retard retinal degeneration and be a promising therapeutic target to treat various retinal degenerative disorders.

## Introduction

Inherited retinal degeneration (IRD) comprises a heterogeneous group of ocular diseases caused by mutations in more than 260 genes identified to date. The disease manifests as degeneration of rods (e.g., retinitis pigmentosa) and cones (cone–rod dystrophy), primarily due to mutations in rod- or cone-specific genes. This occurs in addition to mutations in genes expressed in retinal pigment epithelial (RPE) cells, which lead to photoreceptor degeneration (e.g., Leber’s congenital amaurosis [LCA]) [1].

Previous work identified common signaling pathways in retinal degeneration in various animal models regardless of the affected genes or mutations. The study also indicated that reprogramming a metabolism towards anabolic processes for biosynthesis may be a novel therapeutic strategy for photoreceptor neuroprotection during acute stress [2]. Therefore, strategies that boost the anabolic rate and meet photoreceptor demands during retinal degeneration could generate a therapeutic platform independent of affected genes and mutations.

One common pathway affected in degenerated retinas is the altered protein kinase B (AKT)/mammalian target of the rapamycin (mTOR) axis. A serine/threonine protein kinase mTOR exists in distinct mTORC1 and mTORC2 complexes. Together with AKT, mTOR plays a critical role in a wide spectrum of cellular signaling, including regulation of autophagy, lysosome biogenesis, energy metabolism, protein and lipid synthesis, cytoskeleton organization, and cell survival. Debates continue on the therapeutic targeting of the AKT/mTOR axis under conditions associated with aging, neurodegenerative disorders, cancer, and diabetes, and whether the activation or inhibition of mTOR should be taken as a therapeutic approach is currently under investigation. While researchers are still searching for an answer to this question, they agree that the severity, the stage of the disease, and the variety of affected tissues and cells should be taken into consideration in solving this puzzle. Thus, recent work has reviewed the mTOR signaling pathway, altering it under different neurodegenerative conditions and emphasizing the complexity of mTOR targeting [3]. For example, in Alzheimer’s disease, mTOR inhibition is beneficial for degenerating neuronal cells because this approach leads to stimulation of autophagy necessary for aberrant protein degradation. In contrast, another study on the therapeutic potential of l-DOPA treatment for patients with Parkinson’s disease found that mTOR hyperactivation brings benefits to neuronal cell survival [4, 5].

Another example is a study on Huntington disease (HD), in which a decrease in mTOR activation associated with sequestrated Huntingtin protein (Htt) aggregates in postmortem HD brains was found [6, 7]. Lee and colleagues provided evidence that introducing the constitutively active form of the mTORC1 activator Ras homolog enriched in brain (RHEB) into HD mouse brains alleviates mitochondrial dysfunction, aberrant cholesterol homeostasis, striatal atrophy, and impaired dopamine signaling and increases autophagy.

In the vision science field, mTOR signaling has also generated interest as a therapeutic target. However, similar to the central nervous system neurodegeneration research field, there is a lack of consensus on the therapeutic targeting of mTOR for different retinal degenerative disorders. Moreover, it has been demonstrated that depending on the insult, the same retinal cell type could respond to the same treatment in a different way. For example, the death of retinal ganglion cells (RGC) known to occur through apoptosis during the course of glaucoma and diabetic retinopathy is differently regulated by mTOR-mediated autophagy, and the role of autophagy may differ depending on the triggering injury and the relevant cell death pathway [8]. Phosphorylated mTOR (p-mTOR) is significantly downregulated in both diabetic and glaucomatous retinas [8]. However, depending on whether or not p-AMPK is involved in the retinal pathogenesis, the results of mTOR-mediated regulation of autophagy can vary. Chronic intraocular pressure elevation results in autophagic cell death without AMRK involvement, which is not the case for the changes in the diabetic retina [8]. Therefore, in this study, the authors concluded that IOP elevation was associated with autophagy activation, resulting in RGC death, while in the diabetic retina, the AMPK-mediated autophagy induction may serve as a survival attempt of RGCs.

Indeed, multiple studies with animal and cellular models of different retinal degenerative diseases have revealed that the targeting of mTOR does not lead to a straightforward cell fate. An investigation conducted with RPE cells deficient in oxidative phosphorylation, mimicking molecular events occurring in age-related macular degeneration (AMD), demonstrated that the early RPE changes associated with hypertrophy and dedifferentiation coincide with robust activation of the mTOR pathway [9]. Therefore, to combat AMD, it is necessary to attempt to downregulate mTOR activity. Overall, these results confirmed a previous finding demonstrating that downregulation of mTORC could delay the aging process in the RPE cells [10] and enhance cellular degradation and self-renewal [11]. In mice with oxygen-induced retinopathy, modeling aberrant retinal vascular development, targeting mTOR activity through either rapamycin or VEGF-mediated signaling revealed that, in endothelial cells, the mTOR pathway is activated by VEGF. This finding also demonstrated that mTOR contributes to physiologic vascular development and is modulated in a postnatal, age-dependent manner [12].

Despite the convincing data on the therapeutic effect of mTOR downregulation in these studies, another line of research proposes that mTOR activation could be neuroprotective for degenerating retinal cells. The group led by Dr. Rajala demonstrated that a constitutively active version of mTOR mutant promotes cone survival in mice with RP in a nutrition-independent manner [13]. This study confirmed previous findings from Cepko’s and Punzo’s laboratories indicating that cone cell death in mice with IRD occurs as a result of the downregulation of insulin/mTOR signaling. These laboratories demonstrated that the cell-autonomous activation of mTORC1 delays photoreceptor cell death in animal models of retinal degeneration [14–17]. In support of these findings, a recent study conducted with the tuberous sclerosis complex 1 (*Tsc1*) gene, an mTOR inhibitor, demonstrated that silencing this gene enhanced photoreceptor survival and increased retinal function in PDE6b^H620Q/H620Q^ mice [18].

Despite the significance of the studies conducted with animal models of ocular degenerative diseases, the answer to questions about whether a uniform strategy could be applied in a variety of IRD models remains elusive. Therefore, the disagreement about the role of mTOR in controlling IRD fueled our interest in this molecule in *rd16* mice, mimicking LCA in humans.

In our study, the *rd16* mice demonstrated severe retinal degeneration accompanied by p-AKT/p-mTOR downregulation at P15 [19]. The role of p-AKT/p-mTOR in the degenerating retinas can be studied by modulating its expression, activity, or degradation. Although mTOR inhibitors have been recently identified due to their ability to reduce proliferation in cancers, the development of mTORC1 activators that are beneficial for retinal degeneration [14–17] or other diseases is still a critical need. While many commercially available mTOR inhibitors, including rapamycin, dactolisib, everolimus, etc., are on the market, MHY1485 is the only current potent cell-permeable activator, whose specificity and efficiency in agonizing mTOR activity in vivo still need to be determined. Therefore, we decided to take a genetic approach to reprogram the AKT/mTOR signaling in *rd16* mouse photoreceptors by ablating a pseudokinase tribbles homolog 3 (TRB3 or TRIB3), a protein kinase-like scaffold with impaired catalytic activity, known to inhibit phosphorylation of AKT [20] and mTOR [21].

Integrated stress response (ISR) that is persistently activated in *rd16* mice [19] has been reported to induce TRB3 overexpression via ATF4 [22]. TRB3 participates in a variety of cellular signaling. Thus, TRB3 has been proposed to regulate cell death, stress responses, inflammation, cell differentiation, and protein degradation [23]. It also functions by controlling autophagy flux and protein degradation [23]. TRB3 acts as a metabolic switcher controlling GLUT1 activity and glucose uptake [24]. Therefore, we decided to investigate the TRB3-mediated control of AKT/mTOR signaling in the retina of mice with IRD. We demonstrated that the restoration of the AKT/mTOR axis slows down IRD progression and delays photoreceptor cell death. To our knowledge, this is the first study that has validated TRB3 as a therapeutic target in animal models of retinal degeneration; this study emphasizes the critical need to generate a TRB3-based cellular therapy to retard inherited retinal dystrophies.

## Material and methods

### Animals

BXD24/TyJ-Cep290rd16/J (*rd16*) and C57BL/6J mice were obtained from Jackson Laboratory (Bar Harbor, ME). TRB3^*−/−*^ mice were generated as previously described [25]. All mice used in the study experiments were on C575BL/6 background. The *rd16* mice (Jackson Laboratory) were crossed with TRB3^*−/−*^ mice to produce *rd16* TRB3^*−/−*^ genotype-carrying mice. The mice were housed in the UAB animal core facility with a 12 h light/dark cycle and free access to a standard diet and water. Sample sizes ranged from three to five. Retinas from control and experimental mice of both sexes were used at postnatal days (P) 15, 18, or 20. Mice were euthanized by CO_2_ asphyxiation followed by cervical dislocation. All animal experiments followed a protocol (IACUC#131109793) approved by the University of Alabama at Birmingham Institutional Animal Care and Use Committee (IACUC) and conformed to guidelines from the Association of Research in Vision Science and Ophthalmology.

### Electroretinography (ERG)

ERG was performed using the LKC BIGSHOT ERG instrument. Mice were dark-adapted overnight and were anesthetized with an intra-peritoneal injection of ketamine/xylazine, based on their weight, and placed on a 37 °C heating pad. After pupil dilation with topical 2.5% phenylephrine (Paragon BioTeck, Inc., 42702-102-15), a drop of Gonak solution (AKORN, Lake Forest, IL) was applied, and a monopolar contact loop was placed on the surface of the cornea. Needle electrodes were placed under the scalp and in the tail to serve as a reference and as a ground electrode, respectively. The mice were then exposed to five flashes of 25 cd s/m^2^ in 45 s intervals, and ERGs were recorded (*n* = 3–7). The ERG waveforms were then analyzed using LKC EM software.

### Histology

Eyes were enucleated at P15, 18, or 20 and placed in 4% paraformaldehyde (PFA) for 20 min on ice. After 30 min, a needle (33G) was inserted at the limbus to create a small hole for eye tissue fixation in 4% paraformaldehyde. After 1 h of incubation, the eyes were washed with PBS and immersed in 30% sucrose overnight. The eyes were cryopreserved in an optimal cutting temperature (OCT) compound (VWR: 25608–930) and kept at −80 °C until sectioning. Cryomolds were equilibrated to the temperature inside (−21 °C) the cryostat sectioning system (Leica CM 1510S; Leica, Buffalo Grove, IL, USA). Twelve micromolar retinal sections were prepared using a cryostat tissue sectioning system. The sections were stained with hematoxylin and eosin (Electron Microscopy Sciences: 26754-1A, 26762-01). The nuclei of photoreceptors were then counted by a masked-to-results investigator (*n* = 4–5).

For immunohistochemistry, retinal sections were incubated with primary anti-RHO (1D4) antibody (University of British Columbia) overnight at 4 °C in a humidity chamber, followed by washing and incubation for 1 h at room temperature with secondary antibodies. Sections were counterstained with DAPI (Vector Laboratories, H-1200). Images were collected with an Olympus FluoView-1000 confocal microscope.

To count the red puncta cells in the retinas, the C56BL/6^RFP-GFP-LC3^ mice were crossed with *rd16* and *rd16* TRB3^*−/−*^ to obtain *rd16*^RFP-GFP-LC3^ and *rd16* TRB3^*−*^^/−R^^FP-GFP-LC3^ mice. The puncta become GFP-negative/mRFP-positive (red) upon fusion with lysosomes [26]. At P15, these mice were sacrificed and perfused with 4% PFA, and their eyeballs were nucleated and immersed in 30% sucrose overnight. The next day, the eyeballs were cryopreserved in OCT medium and sectioned to prepare 20-μM retinal sections. Confocal fluorescent microscopy was applied to make the images, focusing on the inner segments (IS) of photoreceptors to count the red puncta. A blind-to-results investigator calculated the red puncta in IS of photoreceptors, evidencing the presence of normally functioning lysosomes with acidic pH (*n* = 5–7).

### Analysis of nascent protein synthesis

The SUnSET method has been described previously [27]. Briefly, mice were intraperitoneally injected with puromycin (puromycin dihydrochloride; Santa Cruz Biotechnology, CAS 58-58-2) at a dosage of 0.04 μmol/g body mass. We then sacrificed the mice and harvested their retinas to prepare protein extracts with RIPA buffer. Total protein (40 μg per well) was electrophoresed on a 4–12% sodium dodecyl sulfate (SDS)-polyacrylamide gradient gel (Bio-Rad, Hercules, CA, USA) and blotted onto a PVDF. The membranes were then incubated with an antibody specific to puromycin (MABE343, mouse) and processed with a secondary antibody specific to IgG2a (goat anti-mouse peroxidase affinipure IgG, Fcγ Subclass 2a Specific: 115-035-206; Jackson Immuno Research Laboratories Inc., West Grove, PA, USA). After scanning the density of the puromycin-incorporated bands using a LI-COR imager, the membranes were stained with Coomassie Brilliant Blue R‐250 staining solution (Bio‐Rad, #1610436) for their normalization. The relative densities of entire lanes were measured using ImageJ software (*n* = 4–6).

### Cell culture, treatment, and transfection

A spontaneously immortalized human Müller cell line (developed by Limb et al. in 2002 [28]) was maintained in DMEM (Hyclone, South Logan, UT), supplemented with 1 mM l-glutamine, FBS (10%; BioAbChem, Ladson, SC), glucose (4.5 g/L), penicillin/streptomycin (100 units/mL; 0.1 mg/mL) in a humidified 5% CO_2_ incubator at 37 °C. Following incubation, the cells were detached by digestion with 0.25% trypsin and continually passaged. Lipofectamine 2000 (Invitrogen, CA, USA) was used for transient transfection according to the manufacturer’s protocol. Briefly, MIO-M1 cells in 12-well plates were transfected with 3 μg of pAAV-CAG-TRIB3-GFP in opti-MEM (Invitrogen, CA, USA). Five hours later, the medium was replaced with fresh cell culture medium containing FBS (10%) and an antibiotic mixture. Experiments were quadruplicated and repeated three times.

Antisense oligodeoxynucleotides (ASO) were synthesized by Sigma-Aldrich. The ASO sequence against TRB3 (ASO-TRB3) was 5′-GTCCAGTCATCACACAGGCA-3′. The control ASO has the sequence 5′-CCTTCCCTGAAGGTTCCTCC-3′ and was introduced into cells as a negative control. The first five bases and last five bases of chimeric ASOs have a 2′-O-(2-methoxy)-ethyl modification. The ASOs were selected based on the data provided by Weismann et al. [29]. For the ASO assay, 12 h after the transfection of MIO-M1 cells with TRB3 cDNA, the cells were transfected with 3 μg of ASO-TRB3 or ASO-control. The cells were collected 48 h after ASO transfection.

### Retinal explants

Retinas were isolated from each animal. Individual right mouse retinas were incubated in culture media with 50 μM chloroquine, while left retinas were incubated without chloroquine for 24 h at 37 °C in a humidified cell culture chamber with 5% CO_2_. After the treatment retinas were washed in PBS, retinal protein extracts were prepared and western blot was conducted. Ratios of LC3II/I and p62 in treated vs. untreated retinas were used to analyze the capacity of the retinas to proceed with LC3 I lipidation (*n* = 6) and accumulate p62 (*n* = 4–5).

### Immunoblotting and immunoprecipitation

The mouse retinas were dissected and lysed with RIPA buffer supplemented with 1% Halt protease and phosphatase inhibitor cocktail (Thermo Fisher Scientific, Waltham, MA). After homogenization, the retina extracts were rotated for 40 min at 4 °C and centrifuged at 14,000*g* for 20 min at 4 °C. The protein concentration of the lysates was estimated using Bio-Rad protein assay (5000001, Hercules, CA, USA). Sample proteins (40–60 μg) were separated by SDS-PAGE and electroblotted to a PVDF membrane. The following antibodies were used: rabbit anti-p-mTor (D9C2, 5536), rabbit anti-mTOR (7C16, 2983), rabbit anti-p-AKT (p-S473, 4060), rabbit anti-AKT (4691), rabbit anti-4E-BP1 (53H11, 9644), rabbit anti-p-4E-BP1 (T37/46, 2855), rabbit anti-Beclin-1 (D40C5, 3495), rabbit anti-LC3A/B (D3U4C, 12741), and rabbit anti-Ubiquitin (3933) from Cell Signaling; rabbit-anti-LAMP2 (L0668), mouse anti-Parkin (05–882), rabbit anti-Actin (A2066), and mouse anti-β-Actin (A2228) from Sigma-Aldrich; rabbit anti-SQSTM1/p62 (ab101266) and rabbit anti-UBR1 (ab42420) from Abcam; and TRIB3 (B-2) sc-390242 and TRB-3 (D-4) sc-365842 from Santa Cruz Biotechnology.

The total extracts from eight retinas were immunoprecipitated with Rho 1D4 monoclonal antibody according to the manufacturer’s procedures. Briefly, the extracts were incubated at 4 °C with 5 µg of antibody or an equal amount of normal mouse IgG (Millipore Corp., USA) overnight and subsequently with protein A/G agarose beads (Santa Cruz Biotechnology) for 4 h. The beads were washed four times with 1× PBS. The immunoprecipitated proteins were eluted for 10 min at 70 °C with 2*Lammie buffer and further investigated by immunoblotting.

### Statistics

The Student’s *t-*test was used to compare two groups, and one-way ANOVA was carried out to compare three or more groups. All statistics were calculated using Graphpad Prism 8 software.

## Results

### *rd16* TRB3^*−/−*^ mice demonstrated an increase in the AKT → mTOR axis activity

mTOR activity is regulated by activated Rheb, growth factors, and amino acid supplementation or by a decrease in the phosphatase activity of the proposed MTMR3 phosphatase, a member of the myotubularin phosphatidylinositol 3‐phosphatase family [30]. We decided to use an alternative approach employing genetic ablation of TRB3 to reprogram the p-AKT and p-mTOR levels; TRB3 has been reported to inhibit the activity of both AKT [20] and mTOR [21].

**Fig. 1:**
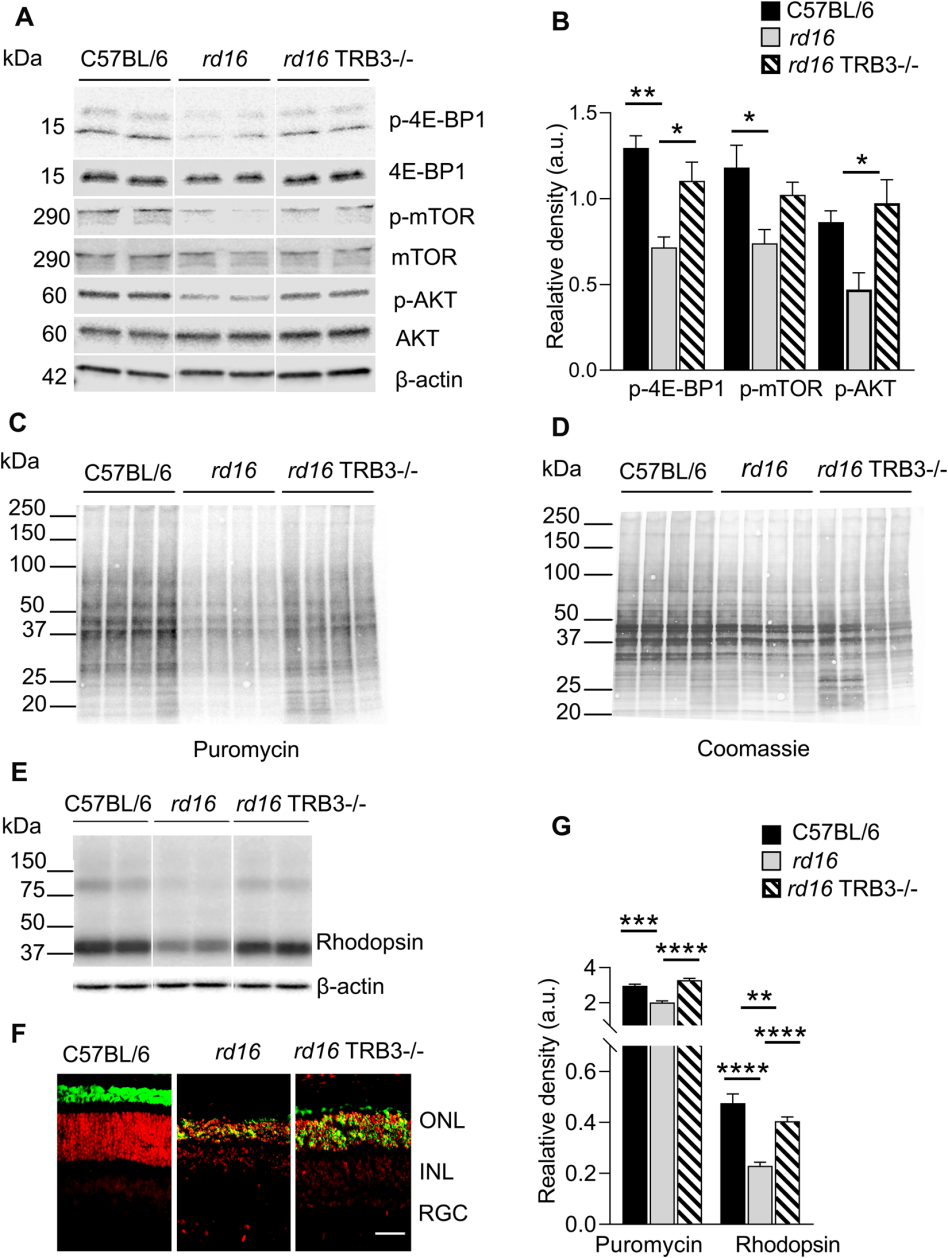
TRB3 ablation restores the p-AKT/p-mTOR axis in *rd16* retinas. **A** Images of western blot membranes treated with anti-p-4E-BP1, -4E-BP1, -p-mTOR, -mTOR, p-AKT^Ser473^, -AKT, and -actin primary antibodies and secondary antibodies scanned with LI-COR imager in three groups of mice. **B** Quantitation of band density of western blot images demonstrates a statistically significant increase in p-4E-BP1 and p-AKT in *rd16* TRB3^−/−^ retinas. Increase in p-4E-BP1 was also accompanied by an enhancement of mTOR activity (*n* = 4). **C** Images of a western blot membrane treated with anti-puromycin antibody to detect the translational level in retinas (*n* = 4). Retinal protein extracts containing incorporated puromycin as the result of intraperitoneal injection in mice were run to detect the density of incorporated puromycin. The density was normalized through protein loading detected by staining with coomassie blue (**D**). **E** Retinal protein extracts from P15 C57BL/6, *rd16*, *rd16* TRB3^−/−^ mice were run on polyacrylamide gel. Western blot images of membranes immunoblotted with anti-Rhodopsin antibody are shown (*n* = 4). **F** Retinal cryosections of P20 C57BL/6, *rd16*, and *rd16* TRB3^−/−^ mice were used to perform the IHC with antibody against rhodopsin (in green). Propidium iodide labels the nuclei of the outer nuclear (ONL) and the inner nuclear (INL) layer, and the retinal ganglion cells (RGC). Accumulation of rhodopsin in the ONL was observed in both *rd16* and *rd16* TRB3^−/−^ retinas. **G** Quantitation of the incorporated puromycin in animal retinas to detect translational level (**C**, **D**) and rhodopsin protein for western immunoblotting. **E** The restoration of the translational rate and an increase in rhodopsin level were observed in *rd16* TRB3^−/−^ retinas (*n* = 4). Data are shown as mean ± SEM; a.u. = arbitrary units; the scale is 50 µm. *p < 0.05, **p < 0.01, ***p < 0.001, and ****p < 0.0001.

The *rd16* mice demonstrated a significant increase in the TRB3 level accompanied by both p-AKT and p-mTOR downregulation at P15–P20 [19]. Therefore, we generated the *rd16* TRB3^*−/−*^ mice to monitor the status of the p-AKT/p-mTOR axis in their retinas. Histological and molecular biological assessments of *rd16* TRB3^−/−^ retinas were conducted from P15 to 18. We learned that TRB3 ablation in degenerating retinas enhances levels of both p-AKT and p-mTOR at P15. Furthermore, while no difference in p-AKT levels was observed between C57BL/6 and *rd16* TRB3^−/−^ retinas (Fig. 1A, B), the decrease in p-AKT level between *rd16* and *rd16* TRB3^−/−^ was dramatic (*p* < 0.05 for comparison between *rd16* and *rd16* TRB3^−/−^). Reduction in p-mTOR was also detected between C57BL/6 and *rd16* retinas confirming previous results [27]. The difference between *rd16* and *rd16* TRB3^−/−^ measured by one-way-ANOVA was not statistically significant.

**Fig. 2:**
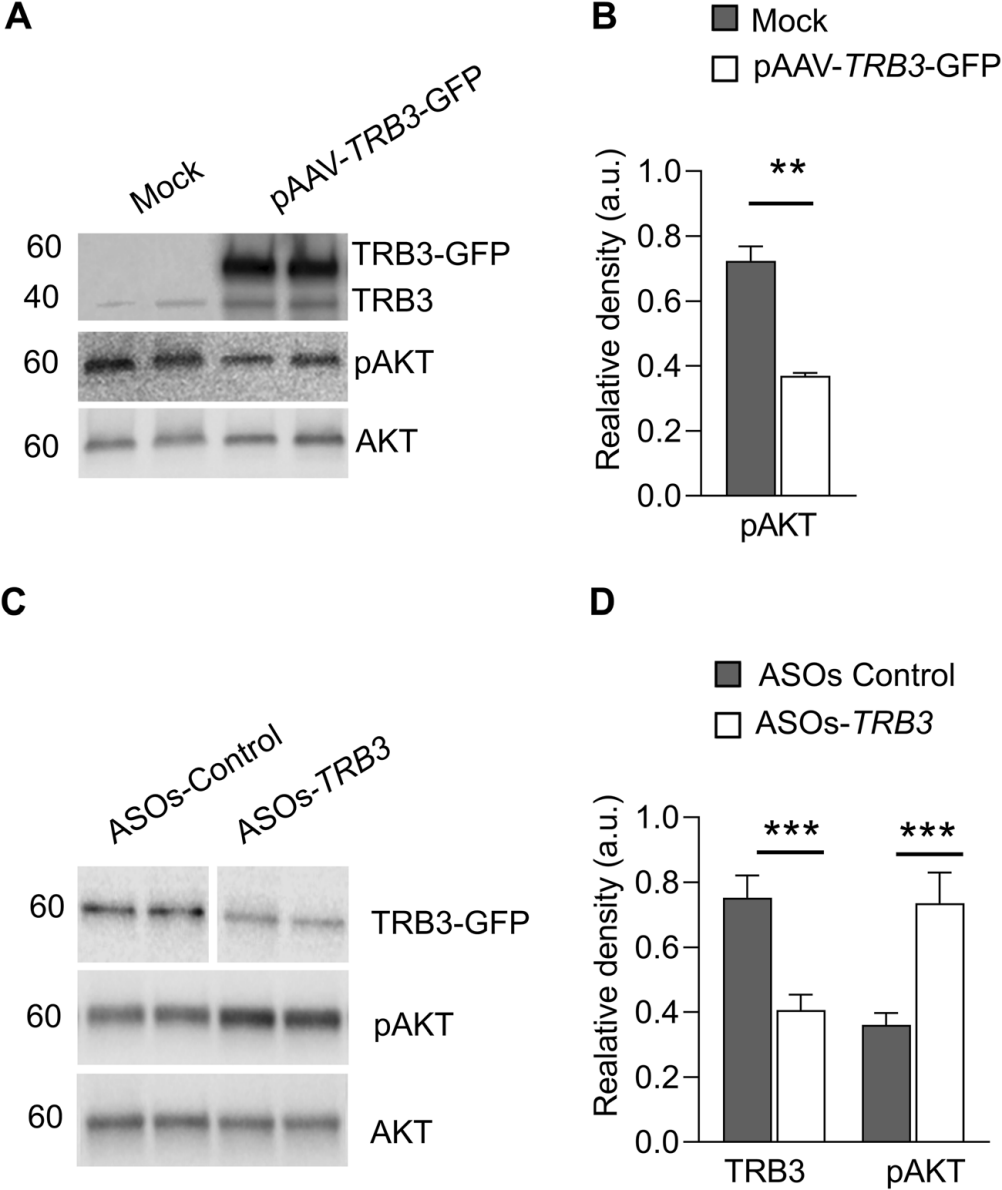
The impact of TRB3 on the phosphorylation of AKT registered in MIO-M1 cells overexpressing TRB3. **A** MIO-M1 cells were transfected with AAV-CAG-TRB3-GFP plasmid. Seventy-two hours post-transfection, the cells were harvested, and protein extracts were separated running polyacrylamide gel. Images of western blots stained with anti-TRB3 and anti-pAKT^Ser473^ antibodies are shown. **B** Quantitation of p-AKT normalized to AKT is shown. About a 50% reduction in p-AKT level was observed (*n* = 4). **C** MIO-M1 cells were transfected with AAV-CAG-TRB3-GFP plasmid in addition to control and anti-TRB3 ASOs for 48 h. TRB3, p-AKT, and AKT were detected in the protein extracts. **D** An analysis of densitometry of TRB3 and pAKT^Ser473^ bands normalized to the bands for β-actin and AKT, respectively, is shown (*n* = 4). Data are shown as mean ± SEM; a.u. = arbitrary units; **p < 0.01, ***p < 0.001.

To confirm the effect of TRB3 on AKT phosphorylation, we next performed an in vitro study with MIO-M1 Müller glial cells. In these cells, we overexpressed mouse TRB3 cDNA and observed a significant 50% reduction in the p-AKT^S473^ level (Fig. 2A, B). Moreover, when we treated the MIO-M1 cells overexpressing TRB3 with ASO-TRB3, a dramatic reduction in TRB3 level accompanied by the restoration of p-AKT^S473^ was detected (Fig. 2C, D). These results suggest that in retinal tissues, TRB3 can regulate AKT activity.

### TRB3 ablation in *rd16* mouse retinas reduced photoreceptor cell death and delayed retinal degeneration

**Fig. 3:**
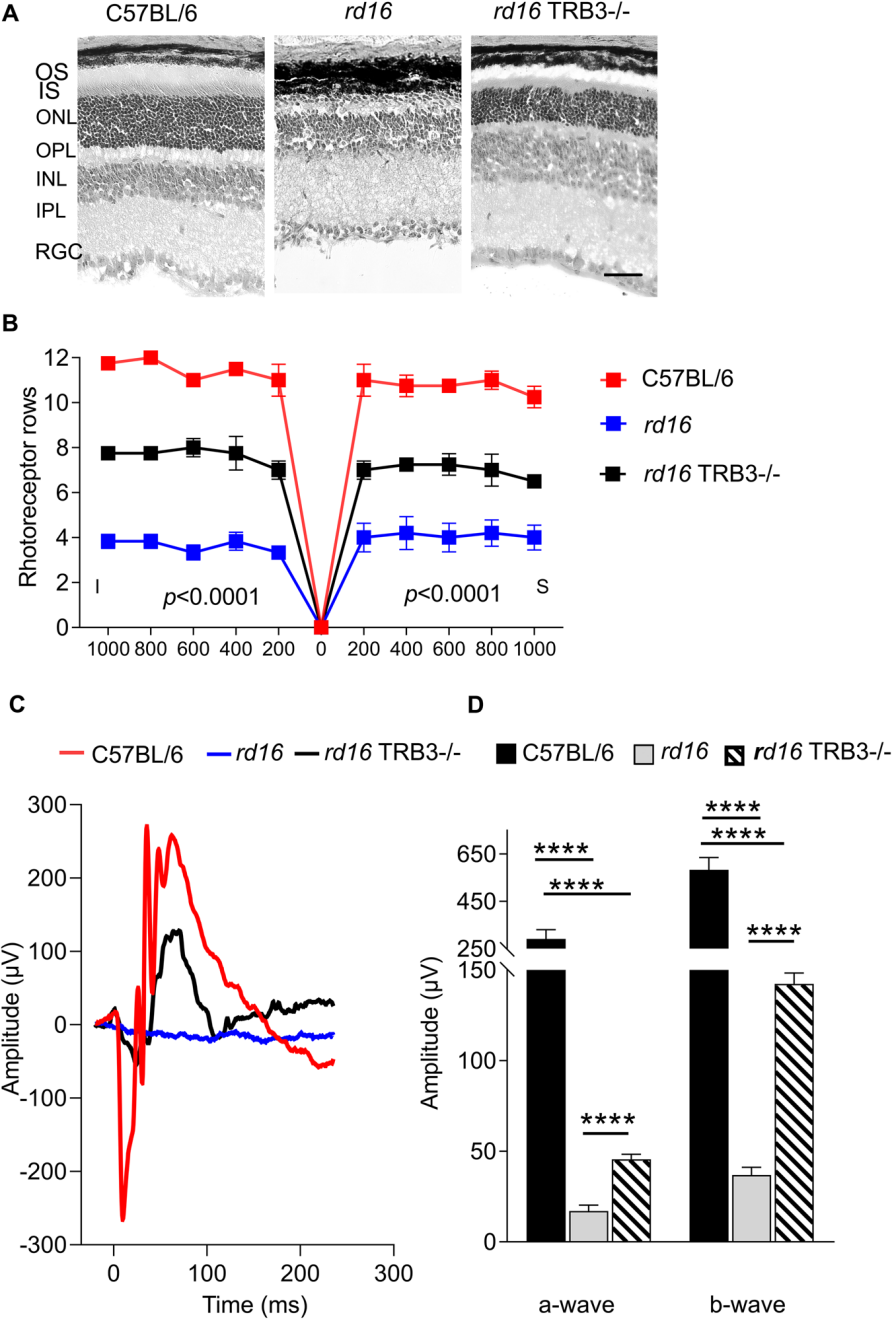
TRB3 knockout decreases the photoreceptor cell loss detected in P18 *rd16* retinas. **A** Images of C57BL/6, *rd16*, and *rd16* TRB3^−/−^ retinas stained with eosin and hematoxylin are shown. The scale is 50 µm. **B** The retinal images were used to calculate the photoreceptor nuclei across the retinas. Preservation of photoreceptor cells was detected in *rd16* TRB3^−/−^ mice compared to *rd16* mice (*n* = 4–5). This preservation was in accordance with the improvement of a- and b-scotopic ERG amplitudes registered at P18 (*n* = 3–7). **C** Images of the scotopic ERG traces. **D** ERG analysis demonstrated a statistically significant increase in the a- and b-wave amplitudes in *rd16* TRB3^−/−^ retinas. Data are shown as mean ± SEM; ****p < 0.0001. OS outer segments of photoreceptors, IS inner segments of photoreceptors, ONL outer nuclear layer, OPL outer plexiform layer, INL inner nuclear layer, IPL inner plexiform layer, RGC layer retinal ganglion cell layer.

Histological analyses of *rd16* TRB3^−/−^ retinas demonstrated that TRB3 ablation delayed the death of photoreceptor cells; four more rows of photoreceptor cell nuclei were detected across the retina on average (*p* < 0.0001) at P18 (Fig. 3A, B). The retardation of the death of photoreceptor cells in *rd16* TRB3^−/−^ retinas was also accompanied by increases in the scotopic a- and b-wave ERG amplitudes (*p* < 0.0001). We detected a twofold increase in the a-wave and a fourfold increase in the b-wave amplitudes at P18 (Fig. 3B–D). Altogether, these data suggest that TRB3 ablation delays retinal degeneration in *rd16* mice. Therefore, we next decided to analyze whether the recovery of the AKT/mTOR pathway affects downstream signaling in degenerating photoreceptors.

### *rd16* TRB3^−/−^ mice manifested a restoration of translational rate and an increase in rhodopsin level

AKT/mTOR signaling is known to participate in a variety of cellular responses, including metabolism and autophagy. Therefore, we next assessed the translation rate known to be attenuated in P15 *rd16* retinas [19]. Because 4E-BP1 is known to be phosphorylated by mTORC1 [31], we then decided to check whether the levels of p-4E-BP1 is altered in *rd16* TRB3^−/−^. We found that the *rd16* TRB3^−/−^ mouse retinas manifested an increase in the p-4E-BP1 level (Fig. 1A, B). This increase implies that the *rd16* TRB3^−/−^ degenerating retinas could have a diminished level of active translational repressor, which interfered with the assembly of the 5-cap mRNA recognition complex. Therefore, using the puromycin-based SUnSET method previously validated in the retina [19], we further learned that the rate of protein synthesis was restored in *rd16* TRB3^−/−^ retinas compared to *rd16* and C57BL/6 retinas (Fig. 1C, D, G, *p* < 0.001). Because of translational restoration and the fact that rhodopsin is the most abundant retinal protein, we next assessed the rhodopsin level in degenerating retinas. We found that a dramatic increase in rhodopsin protein occurred along with the increase in the general translational rate in *rd16* TRB3^−/−^ retinas (Fig. 1E–G, *p* < 0.001).

### *rd16* TRB3−/− retinas manifested restoration of autophagy-associated protein expression and improved autophagy flux

**Fig. 4:**
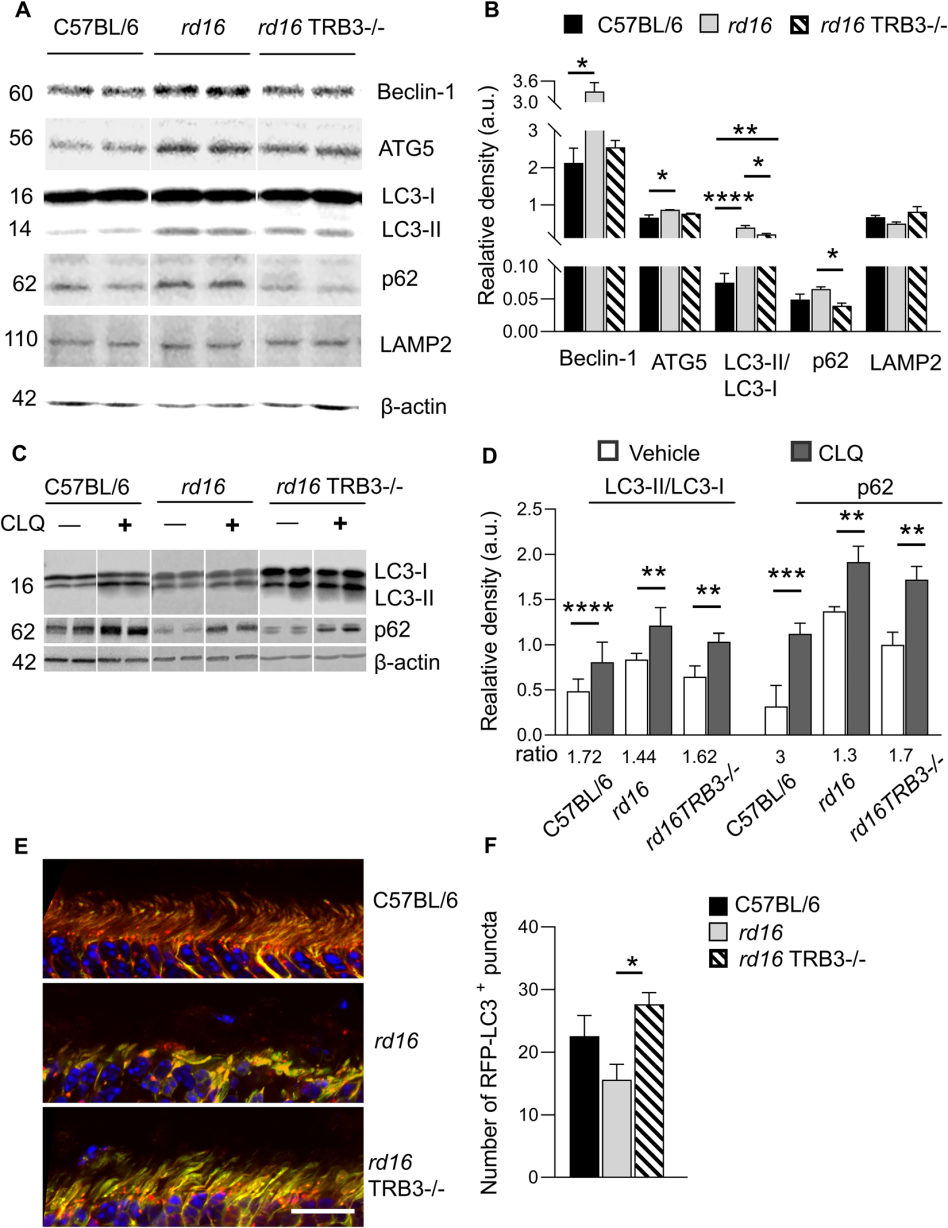
TRB3 ablation in P15 *rd16* retinas resulted in restoration of autophagy-associated protein levels and flux. **A** Images of western blot membranes probed with anti-Beclin-1, ATG5, LC3, p62, and LAMP2 antibodies. **B** Quantitation of Beclin-1, ATG5, LC3 II/I, p62, and LAMP2 in protein retinal extracts from three groups of mice (*n* = 4–5). **C**, **D** Treatment of retinal explants with chloroquine (CLQ), a classic inhibitor of autophagy, was used to determine the capacity of degenerating retinas to promote autophagosome fusion with lysosomes. **C** Images of western blot analysis demonstrated LC3 conversion and p62 accumulation. **D** Quantitation of LC3 conversion in CLQ-treated retinas (*n* = 6). The LC3 II/I ratios of the treated retinas were normalized to those of untreated retinas. An increase in induced LC3 conversion was detected in the treated C57BL/6 and *rd16* TRB3^−/−^ retinas. We also observed an increase in p62 level in treated and normalized *rd16* retinas as compared *to* C57BL/6 and *rd16* TRB3^−/−^ (*n* = 4–5). **E**, **F** The RFP-GFP-LC3 transgene expression in mouse retinas demonstrated the generation of red puncta in the inner segments of photoreceptors. **E** Images of the retinal sections of C56BL/6, *rd16*, and *rd16* TRB3^−/−^ mice taken with a confocal microscope. **F** Quantitation of the red puncta cells in the retinal sections of the three groups of mice (*n* = 5–7). Data are shown as mean ± SEM; *p < 0.05, **p < 0.01, ***p < 0.001, and ****p < 0.0001.

Autophagy regulated by mTOR is a major digestion process that removes damaged macromolecules and organelles in cells [32]. To learn about how mTOR activity is boosted in *rd16* TRB3^*−/−*^ retinas, we initiated a comparative study to evaluate autophagy-associated protein expression. We found that levels of Beclin-1, a protein involved in the initial step of phagophore formation, increased in *rd16* retinas compared to C57BL/6 and *rd16* TRB3^−/−^ mice (Fig. 4A, B, *p* < 0.05). The same pattern was observed for ATG5 protein in *rd16* retinas when compared to C57BL/6 retinas (*p* < 0.05). Because of the increase in ATG5, a protein influencing the growing phagophore and LC3 integration, we next determined the LC3 processing and measured non-lipidated and lipidated forms. We found that the LC3-II/I ratio was significantly increased in *rd16* mice compared to C57BL/6 and *rd16* TRB3^*−/−*^ retinas (*p* < 0.05), suggesting that LC3 II degradation by autophagosome may be impaired. Furthermore, the level of SQSTM1/p62, a selective autophagy receptor and a link between LC3 and ubiquitinated substrates, was also increased in *rd16* retinas (*p* < 0.05). Together with LC3 II accumulation, the increase in p62 level indicates that autophagy flux may be impaired and should be carefully examined in degenerating retinas. To investigate further, we assessed the level of LAMP2, a lysosome-associated membrane protein-2 and an important regulator of autophagy. We found that *rd16* retinas manifested a reduced level of LAMP2 compared to C57BL/6 mice detected by western blot analysis, but this reduction was not statistically different.

Given that LC3-II/I conversion is increased in *rd16* retinas, we next treated degenerating retinas with chloroquine (CLQ), a classic inhibitor of autophagy, to determine the capacity of autophagosomes in degenerating retinas to bind and deliver LC3 II to lysosomes (Fig. 4C, D). We observed that the LC3 conversion from I to II was limited in the CLQ-treated *rd16* mice, while the treated *rd16* TRB3^−/−^ retinas—although to the degree observed in the C57BL/6 retinas—demonstrated an increase in the conversion step compared to the untreated retinas. Thus, the LC3-II/I ratio in the treated mice compared with the ratio in the untreated C57BL/6 and *rd16* TRB3^−/−^ retinas was 1.73 and 1.62, respectively, compared to *rd16* retinas with a conversion index of only 1.44.

We next identified the p62 accumulation in treated compared with untreated retinas and found that the *rd16* retinas demonstrated an impaired capacity to accumulate p62 under the CLQ treatment (Fig. 4D, E). The ratio of p62 in treated over untreated *rd16* retinas was 1.3, while for C57BL/6 and *rd16* TRB3^−/−^ retinas, the ratios were 3.0 and 1.7, respectively.

Different transgenic mouse models may be leveraged to monitor various stages of autophagosome performance. One of these is the GFP-LC3 transgenic mouse, which has been widely used in the research field of retinal degeneration. In our study, we utilized mice expressing the RFP-GFP-LC3 reporter and conducted fluorescent microscopy at P15 to monitor positive RFP puncta in C57BL/6, *rd16*, and *rd16* TRB3^−/−^ retinas expressing transgene (Fig. 4E, F). The results of the study indicated that the RFP signal, serving as a marker of autolysosomes, was generated in the ISs of photoreceptors. Counting the RFP-positive puncta in the ISs revealed that *rd16* mice had less formed autophagosolysosome fusion compared to *rd16* TRB3^−/−^ photoreceptors (Fig. 4E, F). Altogether, these data indicated an increase in the autophagy-associated protein levels and impaired autophagolysosome function in *rd16* retinas, while an improvement of autophagy flux in *rd16* retinas was observed with TRB3 ablation.

### *rd16* TRB3^−/−^ retinas demonstrated an increase in the level of total protein ubiquitination and E3 ligases

**Fig. 5:**
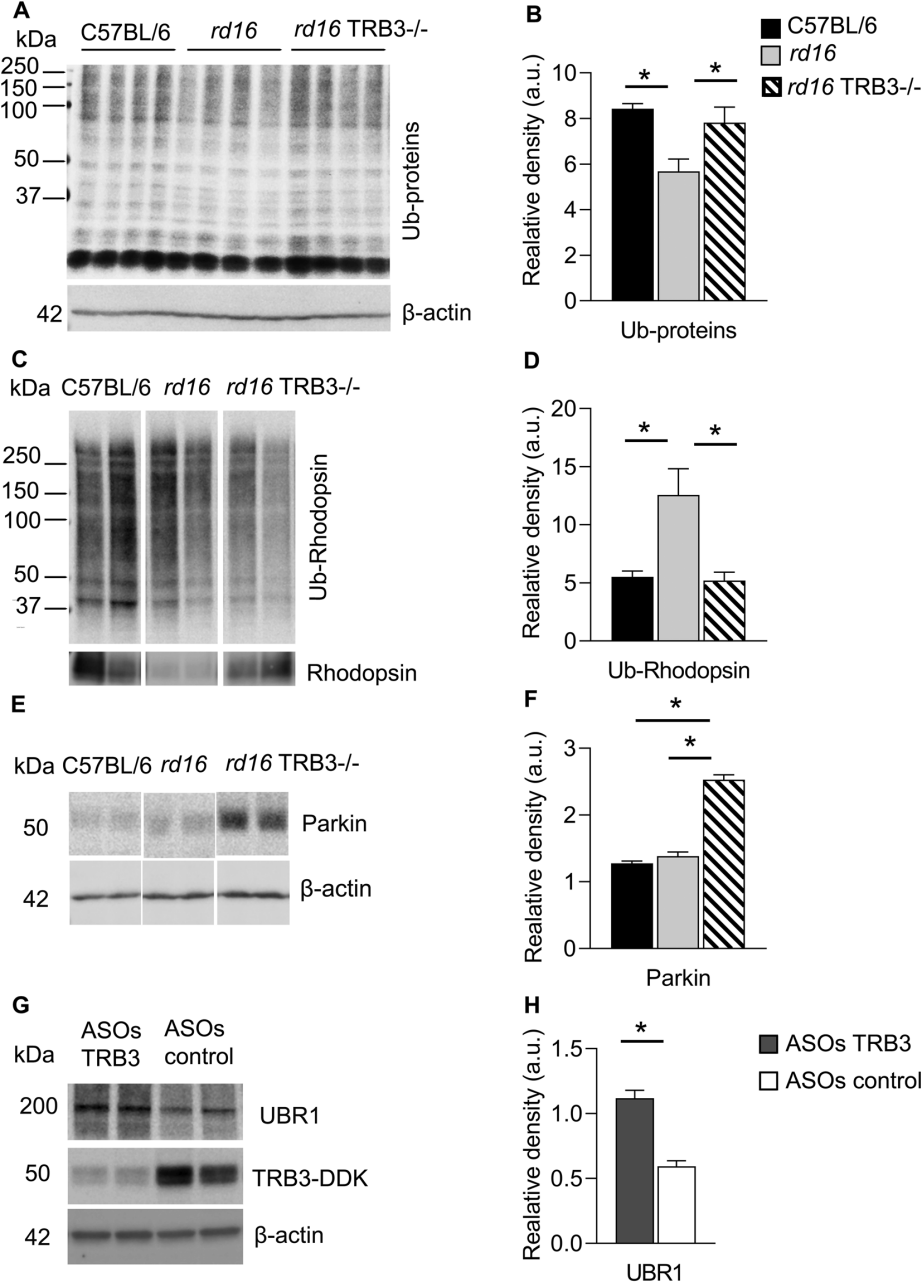
TRB3 ablation in *rd16* retinas led to the restoration of the ubiquitinated protein level and an increase in E3 ligase expression. **A** Images of western blot membrane probed with anti-Ub antibody to detect level of total protein ubiquitination. **B** Calculation of the band density measured for western blot (**A**). Significant reduction in the total Ub-protein levels was detected in *rd16* retinas (*n* = 8). **C**, **D** The Ub-rhodopsin level was detected by performing the IP reaction using protein extracts from C56BL/6, *rd16*, and *rd16* TRB3^−/−^ mice. **C** Images of western blot probed with anti-Ub and Rhodopsin antibodies. **D** Levels of Ub-rhodopsin from IP reactions were normalized through total rhodopsin protein. Measurement indicated that rhodopsin protein was highly ubiquitinated in the *rd16* retinas. **E**, **F** Parkin 1 ligase expression in the retinas of C56BL/6, *rd16*, and *rd16* TRB3^−/−^ mice and MIO-M1 Müller cells (*n* = 4). **E** Images of western blot obtained with retinal protein extracts from P15 C56BL/6, *rd16*, and *rd16* TRB3^−/−^ mice probed with anti-Ubr1, Parkin 1, and β-actin antibodies. **F** TRB3 ablation in *rd16* resulted in increase in Parkin 1 protein compared to both the C57BL/6 and *rd16* mice. **G**, **H** Sequential transfection of Müller cells with plasmid expressing *TRB3* cDNA and ASO-TRB3 (*n* = 4). **G** Images of western blots running with protein extracts probed with anti-UBR1, TRB3, and β-actin antibodies. **H** Quantitation of UBR1 expression in cells co-transfected with ASO-control and ASO-TRB3. A significant decrease in UBR1 level was observed in cells transfected with ASO-TRB3. Data are shown as mean ± SEM; *p < 0.05, **p < 0.01, ***p < 0.001, and ****p < 0.0001.

Recently, it has been highlighted that many neurodegenerative disorders share a common pathological feature, that is, accumulation of intracellular ubiquitin-positive inclusions formed by aggregate-prone neurotoxic proteins [33]. A study conducted in Arshavsky’s laboratory demonstrated that the increase in ubiquitin proteasomal system (UPS) activity supports photoreceptor survival in IRD [34]. Because mTOR inhibition activates overall protein degradation by the UPS system as well as by autophagy [35], we next decided to analyze the level of total ubiquitinated (Ub) proteins. Surprisingly, western blot analysis demonstrated that the level of Ub proteins was reduced in the *rd16* retinas, while in the *rd16* TRB3^−/−^ retinas, it was restored to the level observed in the C57BL/6 retinas (Fig. 5A, B). These results are in accord with the fact that the autophagy flux detected in earlier experiments was impaired in *rd16* retinas; both the selective autophagy and the UPS utilize tagged proteins for further degradation in cells. Additionally, these data made us wonder how a major photoreceptor-specific rhodopsin protein is ubiquitinated and degraded in *rd16* retinas.

A properly folded rhodopsin is known to traffic to the outer segments (OS) of photoreceptors and be degraded by phagocytosis in the RPE cells. This process consists of multiple steps, including recognition by the RPE cells, binding, ingestion, phagosome maturation, and degradation of phagocytosed material [36]. Moreover, the interruption of any of these steps, such as the defect in OS development observed in *rd16* retinas, could lead to the accumulation of unprocessed material inside the retina and eventually to retinal degeneration. As evidence, we observed the accumulation of rhodopsin in the ONL of *rd16* retinas at P20 (Fig. 1F). At P15, we subjected *rd16* retinal extracts to IP with 1D4 antibody and conducted western blot analysis; we found that the Ub-rhodopsin level was significantly increased in *rd16* retinas compared to C57BL/6 and *rd16* TRB3^−/−^ retinas (Fig. 5C, D). Of note is that, in earlier experiments, the same *rd16* retinas (Fig. 1E, G) demonstrated the lowest level of total rhodopsin among the three groups, suggesting the majority of synthetized rhodopsin is ubiquitinated in degenerating retinas. Overall, these findings suggest that mTOR inhibition observed in *rd16* retinas correlates with the increase in the Ub-rhodopsin level. Keeping in mind these facts, we next asked how rhodopsin degrades in *rd16* retinas. We tested the hypothesis that Ub-rhodopsin could be targeted by selective autophagy degradation, and rhodopsin is accumulated in the retina in response to treatment with CQL. We found no difference in the rhodopsin level in retinas treated with autophagy inhibitor (data not shown), noting that that in *rd16* retinas, Ub-rhodopsin degrades through UPS.

Because of the enhanced rhodopsin ubiquitination, we next wondered whether E3 ligases are altered in degenerating retinas. Among all the known ligases present in the retina, we chose to test UBR1, which has been reported to be responsible for rhodopsin degradation [37]. We observed no difference in UBR1 expression in both retinal degenerative groups, suggesting that at P15, mice manifesting ciliopathy, although they have different levels of Ub-rhodopsin, have similar UBR1 levels (data not shown).

Unlike the in vivo results, TRB3 controlled UBR1 expression in the human MIO-M1 cells (Fig. 5G, H). The sequential transfection of cells with plasmid expressing TRB3 and ASO-TRB3 demonstrated that, compared to cells transfected with ASO-control, there was a significant restoration of UBR1. Therefore, the in vitro study indicated a potential TRB3-mediated control of E3 UBR1 ligase expression.

We next analyzed the level of E3 Parkin 1 ligase, which has been reported to be a downstream TRB3 target, the expression of which is inhibited by TRB3 [38] (Fig. 5E, F). Indeed, a dramatic increase in Parkin 1 level was detected in *rd16* deficient in TRB3, while *rd16* retinas maintained the basal level of E3 ligase. Overall, these data indicate that the UPS may operate more efficiently in *rd16* TRB3^−/−^ retinas compared to *rd16* retinas, and this activation occurs concomitantly with improvement in autophagy flux and an increase in protein synthesis in these mice at P15.

## Discussion

ISR is involved in retinal pathogenesis in various retinal degenerative diseases. In this study, we presented evidence that IRS-induced TRB3 overexpression in *rd16* retinas is responsible for the progression of retinal dystrophy. First, we found that TRB3 regulates AKT/mTOR phosphorylation in the retina. Then, we learned that TRB3 ablation is responsible for the restoration of AKT/mTOR activity in these mice. The restoration of p-mTOR results in photoreceptor cell survival assessed by functional and morphological analyses and serves as a homeostatic rheostat of major cellular pathways in the retina.

The pseudokinase TRB3 regulates AKT phosphorylation. Cell culture experiments that employed ASOs targeting TRB3 have demonstrated direct TRB3-mediated control of AKT activity in Müller cells. Both the upregulation of TRB3 and the decline in pAKT and p-mTOR accompanied by a diminished translational rate occurred in *rd16* mice at P15 [19]. Moreover, the level of deactivated (phosphorylated) translational repressor 4E-BP1 controlled by mTOR was lower in *rd16* retinas compared to C57BL/6 mice. Previously, we found that the regulation of the activity of the translational repressor 4E-BP1 plays an essential role in the 5′-cap translational mechanism in degenerating retinas, and the long-lasting translational attenuation during the course of retinal degeneration results in adverse effects on the photoreceptors [27]. In the current study, we observed that the functional and morphological rescue of *rd16* TRB3^−/−^ photoreceptors associated with AKT/mTOR restoration is accompanied by the repair of the translational rate and an increase in rhodopsin protein.

Numerous studies conducted in various in vitro and in vivo systems have demonstrated that mTOR plays a crucial role in regulating autophagy. For example, mTOR is known to inhibit the VPS34 complex, which includes Beclin as a partner [32]. Therefore, it is no surprise that in our study, *rd16* retinas with diminished mTOR showed upregulation of Beclin-1 and ATG5 proteins and, contrarily, *rd16* TRB3^−/−^ retinas with a restored AKT/mTOR axis manifest autophagy protein levels comparable with C57BL/6 retinas. However, it should also be mentioned that *rd16* retinas manifested impaired autophagy flux; the LC3 II and p62 accumulations in *rd16* retinas provided evidence of autophagic dysfunction. The treatment of retinal explants with CQL shows that *rd16* retinas have less “room” for conversion of LC3 I to II, indicating the original problems with LC3 lipidation in forming LC3 II occurring in these mice.

Not only is autophagosome formation impaired in *rd16* retinas but their fusion with lysosomes is affected, as well. We found that the number of RFP-LC3 in photoreceptors’ autolysosomes declined in *rd16* mice and increased in *rd16* TRB3^−/−^ retinas, indicating the TRB3 mediated control of autophagolysosome fusion. Overall, the application of the RFP-GFP-LC3 mice allowed us to count the autophagolysosome fusion in photoreceptors. However, it is worth mentioning that we need to be cautious about leveraging RFP-GFP-LC3 mice expressing mutant photoreceptor-specific genes that lead to the rapid onset of retinal degeneration. P15 *rd16* mice manifest altered photoreceptor cell morphology with shortening of ISs. This fact may introduce an error in the counting of red puncta cells, which suggests that fluorescence microscopy should be conducted when the ISs are well-preserved in the degenerating retina. However, these time points may not correlate with the rate of autophagy flux dysfunction.

The total Ub-protein level is diminished in *rd16* retinas, whereas it is restored in *rd16* TRB3^−/−^ mice. Since both autophagy and the UPS utilize Ub-labeled proteins, and a compensatory balance between their activities exists in mammalian cells to maintain cellular homeostasis [39], *rd16* retinas may clear Ub-protein less efficiently due to diminished autophagosomes and preferential degradation of Ub-protein by UPS. For example, we found that at P15, when *rd16* retinas manifest a low level of rhodopsin, the majority of rhodopsin is ubiquitinated. Given our results evidencing no rhodopsin degradable by autophagy, it seems that UPS activity is sufficient to support photoreceptors at this point, and no rhodopsin accumulation was observed in the retina at P15 [27]. However, at later time points (Fig. 1F), we observed rhodopsin staining in the ONL in *rd16* TRB3^−/−^ retinas, which could be due to a few reasons: (1) an increase in rhodopsin synthesis, (2) mistrafficking of rhodopsin due to the lack of OS of photoreceptors, and (3) accumulation of rhodopsin due to impaired degradation.

TRB3 is known to regulate autophagy and degradation overall. TRB3 interacts with p62 and impedes p62 autophagic/proteasomal degradation [40]; disrupting the TRB3–p62 interaction is known to restore autophagy [41]. Therefore, TRB3-mediated p62 accumulation could be a reason for the impaired autophagolysosome formation in *rd16* retinas. This also explains why the *rd16* TRB3^−/−^ retinas demonstrated a decrease in p62 accumulation. Overall, these data support earlier proposals that TRB3 overexpression constrains autophagy and that TRB3 knockdown induces autophagy flux [42].

The TRB3 molecule has been reported to regulate degradation through association with E3 COP1 [43], Mindbomb [44], and Parkin 1 ligases [45]. In addition, this protein can activate deubiquitinase USP9x [44] expression in the retina [46] and, therefore, reduce the accumulation of Ub-protein in *rd16* retinas. In our study, we found that TRB3 ablation resulted in a dramatic upregulation of E3 ligase Parkin 1 in *rd16* retinas. In MIO-M1 Müller cells, we also found that UBR1 ligase was controlled by TRB3 expression. On the other hand, the inhibition of mTOR in *rd16* retinas could activate the degradation of proteins independent of TRB3 overexpression. Zhao et al. [35] reported that a rapid increase in UPS-mediated proteolysis resulting primarily from mTORC1 inhibition could occur without the requirement of new protein synthesis or key mTOR targets such as S6Ks, 4E-BPs, or Ulks. These data suggest that TRB3-mediated signaling during the course of IRD directly or indirectly inhibits a clearance of Ub-proteins.

In conclusion, we validated TRB3 as a new therapeutic target to delay photoreceptor cell degradation and functional loss. Collectively, these data demonstrate that TRB3 regulates the AKT/mTOR axis in the retina and impacts AKT/mTOR downstream signaling, including 5′cap-dependent translation, autophagy, and UPS-dependent degradation. Therefore, approaches based on the application of ASOs or interference RNAs as well as on cell penetrating peptides to reduce either the expression or activity of TRB3 should be taken into consideration in the treatment of IRD.
